# Spike-timing theory of working

**DOI:** 10.1186/1471-2202-12-S1-O3

**Published:** 2011-07-18

**Authors:** Botond Szatmáry, Eugene M Izhikevich

**Affiliations:** 1 Brain Corporation, San Diego, CA 92121, USA

## 

It is commonly assumed that long-term memory is represented by patterns of synaptic connections within groups of neurons and that memory recall corresponds to an activation of a group triggered, e.g., by a sensory stimulus. Sustained spiking activity of one or a few selected long-term memory representations is believed to be the neural correlate of working memory.

The capacity of working memory is referred to as being the number of neuronal groups that could be maintained active at the same time. This short-term memory capacity is, for example, thought to be seven plus or minus two items for ordered lists. We distinguish the short-term capacity from long-term memory capacity or repertoire, which is the large number of neuronal groups required to store many distinct memories formed by past sensory experience.

We propose [1] a mechanism for working memory that allows for a greatly expanded long-term memory capacity, and we demonstrate how this mechanism can simultaneously maintain in working memory a few items out of this huge repertoire (which is far greater than the number of neurons).

We assume that (i) the groups of neurons representing long-term memories are largely overlapping and (ii) neurons (within a group) are capable of exhibiting precise firing patterns, so different representations are distinguished not only by which neurons fired, but also by their exact spiking patterns. This is in contrast with previously suggested mechanisms of working memory, where the spike-timing nature of neuronal activity is ignored and the models’ explanatory power is limited to systems having small repertoires of long-term memories represented by, e.g., carefully selected non-overlapping populations of neurons.

Using associative short-term synaptic plasticity in the form of short-term STDP, we demonstrate that a few neuronal groups can be simultaneously selected to transiently be part of working memory, i.e., show elevated and precise firing activity for seconds after initial activation.

Our theory explains the relationship between precise spikes and slowly changing firing rates of neurons engaged in active maintenance of working memory, and it points to the connection between working memory and perception of elapsed time on the order of seconds.
